# Melanotic Sarcoma—A Case1Read at the forty-second annual meeting of the Medical Association of Central New York, held at Auburn, October 19, 1909.

**Published:** 1910-03

**Authors:** W. L. Wallace

**Affiliations:** Syracuse, New York


### Melanotic Sarcoma—A Case[1]

By W. L. WALLACE, M. D., Syracuse, New York.

THE case of melanotic sarcoma which I report is striking because the primary growth was not even suspected until autopsy. Melanotic sarcomata arise from the pigmented tissues of the skin and eye. They usually occur between the fortieth and sixtieth years of life, but have been found as early as fifteen, and as late as eighty-four years of age. They are particularly liable to start from birthmarks, moles, or warts. They may also arise around the nails on the fingers or toes, or in the dark skin of the external genitals or anus; or they may arise from the skin at any place, as at the heel from an injury by a nail in a boot.

The commonest situation of all is the choroid coat of the eye. These eye tumors tend to spread out through the sclerotic coat or anteriorly through the cornea, or back along the optic

---

1. Read at the forty-second annual meeting of the Medical Association of Central New York, held at Auburn, October 19, 1909.

nerve. They are very apt to recur after removal and the secondary growths appear very early. Death is generally caused in less than three years by the growth of secondary deposits. Secondary growths, however, have been known to appear very late, even eleven years after the removal of the eye. Nevertheless, many successful operations have been recorded due to early enucleation of the eye. The glands near the orbit are rarely affected. The most frequent place to find secondary deposits is the liver, and in some cases they are limited to this organ. Often they are widely scattered through all the organs.

John H., a carpenter, aged 46, sent into Hospital of the Good Shepherd, Syracuse, N. Y., June 18, 1909, with the following history. Father died at 82 of kidney trouble. Mother died at 73 of pneumonia. No growths in family. Had been absolutely well so far as he knew until six weeks before, when he noticed a soreness high up under the ribs on the right side, which he attributed to rheumatism. He soon commenced rapidly to lose weight and strength. He had no pain and noticed nothing except the enlargement of abdomen.

The examination showed a man greatly emaciated, with an enormously enlarged liver. No ascites or jaundice were present. The white blood-count was 9,800—80 per cent. polynuclears. The stomach examination revealed the absence of HCl. The diagnosis was—probable cancer of liver, most likely secondary to intestinal or stomach cancer.

Death occurred two days after admission to hospital. An autopsy was performed by Dr. Steensland who found a smooth liver weighing about 28 pounds, covered and filled with small black, white and red spots. On seeing this "granite liver," Dr. Steensland immediately examined the eyes and discovered a small black speck at the junction of the cornea and conjunctiva in the right eye, which he pronounced the primary melanotic sarcoma. Many other secondary growths were found in the peritoneum, heart and lungs. The spleen was not enlarged and there was no ascites. The growth in the eye was extremely small, and careful inquiry proved that he had never complained of any trouble with his eyes.

It would have been pleasant to have discovered the speck in the eye and associated it with the liver before death. It might sometime be a satisfaction at an autopsy to remember that a "granite liver" indicates a primary growth, probably in the eye.

---

E. M. Foote of New York, says that if an aspirator is not at hand an exploring needle may be used for tapping the pleural cavity. The patient may be so placed that the serum will all drip away without using an aspirator. Joint aspiration may be done in the same manner.—*Medical Record*, February 5, 1910.

